# Editorial viewpoints of scientific publishing for early-career research scientists

**DOI:** 10.1186/s12919-023-00286-7

**Published:** 2024-01-16

**Authors:** Pei-Chen Peng, Fadie T. Coleman

**Affiliations:** 1 Department of Computational Biomedicine, Cedars-Sinai Medical Center, Los Angeles, CA 90048 USA; 2 Harvard School of Dental Medicine, Boston, MA 02115 USA

**Keywords:** Scientific writing, Journal submission, Career development, Early-career scientists

## Abstract

While the structure and composition of the scientific manuscript is well known within scientific communities, insider knowledge such as the tricks of the trade and editorial viewpoints of scientific publishing are often less known to early-career research scientists. This article focuses on the key aspects of scientific publishing, including tips for success geared towards senior postdocs and junior faculty. It also highlights important considerations for getting manuscripts published in an efficient and successful manner.

## Background

“Publish or perish” is the cliché often cited as a requirement for success in academia. General resources for scientific publishing are widely available in articles [1–4], books [5–7], and workshops. Previous articles have described the fundamentals of scientific publishing such as a clear abstract [3], a well-structured manuscript [1, 4], and reasonable responses to reviewers’ comments [2]. These articles usually targeted the general academic audience. However, scientific publishing is especially challenging for early-career research scientists, who are in training or have just established an independent investigator role and have limited experience compared to their senior counterparts. Here, we present advice on scientific publishing for postdoctoral fellows and assistant professors. This report provides key insights and highlights gleaned from the 2019 and 2020 NIH-ASCB Accomplishing Career Transition (ACT) webinars, “Scientific Publishing”, led by Dr. Matthew Welch (editor-in-chief of *Molecular Biology of the Cell* (*MBoC*)) and Dr. Jodi Nunnari (editor-in-chief of the *Journal of Cell Biology* (*JCB*)). The ACT program is funded by National Institute of General Medical Sciences Innovative Programs to Enhance Research Training (IPERT) initiative and led by the American Society for Cell Biology (ASCB), which supports professional development and training for postdocs and assistant professors in the biological and biomedical sciences.

### Scientific publishing: a strategic plan for early-career research scientists

Scientific publishing can fulfill several vital purposes for early-career research scientists. For example, high-quality papers receive recognition from the scientific community; peer-reviewed publications establish a track record that can support funding requests such as grant applications; and corresponding authorship can serve as evidence of mentorship and leadership in the tenure-promotion package. While early-career research scientists also juggle other academic responsibilities, scientific publishing is important for moving one’s career forward. Therefore, a targeted plan for scientific publishing is needed and should not be left to chance. In the following paragraphs, we share perspectives and a step-by-step guide for scientific publishing, including a discussion of when to write a paper, how to write a paper, and what to consider after submission (Table 1).

**Table 1:** Checklist questions to ask when writing a scientific paper

When to write?
What are your goals for publishing?
Would you like to present the results in a conference before submission?
How to write?
Are results presented in a logical order?
Can figures be interpreted easily?
Is data reproducible?
What to consider for submission?
Would you like to submit to a preprint server?
Would you like to submit to a general-interest or specialized journal?
What is the quality of the review process? What is the turnaround time?
Who is on the editorial board?

### How can I tell when it’s time to write my paper?

One way of answering this question is to provide the following conceptual framework—think about writing as a continuous process. In other words, write as you go [8]. This way of thinking can help facilitate paying close attention to the many factors that can influence the timing of the writing process. It can be helpful to keep in mind the goals for publishing (e.g. disseminating scientific information, informing the community, and contributing to the field) as you prepare to write [9, 10]. Having a clear objective can help you create a blueprint or outline of your study and future manuscript, that would include ideal stopping points for experimentation, good starting points for writing, and critical check points to monitor progress and assess unanticipated discoveries [9].

A useful exercise toward this goal is the preparation of conference abstracts. The abstract is an effective way of outlining your paper because it allows you to visualize what type of experiments you will need to conduct and where they will fit into the overall big picture. Another approach is to just plunge right in and write up a rough draft of the paper using an approach that prioritizes sketching out figures first and then continues with drafting summaries that discuss the study’s potential contributions and impact to the scientific community [11].

Successful scientific writing involves searching the existing literature to identify knowledge gaps, discerning how your work fits into the current body of science, and designing innovative approaches that will facilitate the collection of compelling results. Therefore, writing as you go can serve as both a guide and promise of something tangible to share with the larger scientific community [10]. The task of writing a compelling story and determining how to best tailor your message to an intended audience can be greatly enhanced by adopting a consistent practice of writing while conducting experiments.

### Fundamentals of writing a paper: organization, data, and figures

The quality of a scientific paper is the most important indicator for journal editors in deciding whether to move forward with the peer review process. Three major aspects contribute to the quality of a scientific paper: organization, data, and figures.

Developing a structure ensures the most impactful findings are discussed and support a logical flow of information throughout the paper. The most common reason for rejection in the initial editorial screening process is not clearly stating the importance of both the research question and conclusions. For clearly stating the importance, a practical writing technique is to organize major points in a logical rather than chronological manner [1]. Presenting your work in a more question-driven fashion can increase the chances of your manuscript being received favorably. A common pitfall is to reiterate the train of thoughts you went through when performing the experiments. It is ideal to have a key message weaving through the narrative and bringing all the different findings identified together. Additionally, publishing all relevant results, rather than selectively choosing only those that support the study’s conclusion, is also an important ethical practice.

The next key is to accurately describe the data. Reviewers carefully assess data reproducibility, depth, and quantification. The authors are advised to pay particular attention to describing data collection and validation, as well as what statistical tests were performed, how controls were designed, and whether quantitative analyses support the claims. The methods should be described in detail so that others can reproduce exactly what the manuscript identified. In addition, the editors noted that it has been increasingly critical to report reproducible figures.

Last, clearly presenting the data is essential in preparing a manuscript. Data visualization is fundamental in modern science. Tutorials, papers [12, 13], and online tools [14] are available for researchers to learn how to clearly present scientific observations. Building a pipeline that generates figures alongside the experiments helps regenerate figures multiple times as you fine tune the experiments. Another useful tip is to start from the small, generating one figure at a time.

### Getting your paper published: key consideration for submission

Adopting a forward-thinking approach to preparing your manuscript can also be helpful with the publishing process. Below, we discussed five things to be considered when submitting a manuscript.

#### Thinking of your audience

An efficient strategy of writing is to keep in mind what editors and peer reviewers look for in a publication. Thinking about how your paper will be read and reviewed at the start of the writing process can help provide perspective around writing style, topic focus, and audience. Keeping such things in mind can also help determine appropriate or target journals for submission [15]. Looking up members of the editorial boards of the journals that you are considering can also be helpful in identifying audiences with common interests and expertise.

#### Deciding on where to submit

In addition to thinking about journals in terms of those that share similar interest areas, one could also consider the overall mission or function of the publication when deciding where to submit your work. For example, submitting manuscripts as a preprint is both encouraged and a great way to get your work to be visible to a wide audience. Preprints are also acceptable forms of publication for grant submissions and job applications. Noteworthy caveats include understanding that the preprint’s open-source nature does call for attention to sensitive data and scientific uncertainty [16, 17]. Should one consider general-interest or specialized journals is another question that is often asked when deciding on where to submit manuscripts. This is an important question and merits careful consideration as it can be a career-shaping opportunity for trainees and junior faculty. Quality of the review process is another factor to consider, which may influence graduation, tenure promotion or mental health. General-interest journals such as *Science*, *Nature*, and *Cell* that have professional editors tend to take longer from submission to publication. The reviewers and editors often require time consuming additional experimentation as a condition for publication. Specialized journals who have practicing scientists serving as the editors have shorter turnaround time. Items of lesser importance would be impact factor [18], as this citation-based metric may not be the most accurate indicator of quality publications [19].

#### Telling a compelling story

As you think about how to best present a compelling story and tailor your message to an intended audience, you can also think about the type of journal that could help you best reach that intended audience. Additional details to consider include: Think about who needs to know the key finding(s): whether it’s a breakthrough that the broad community will be excited about, or a fundamental mechanism that will benefit expertise in the field.

#### Communicating with journals

Now that you have decided to submit to a particular journal, additional questions most likely revolve around how one communicates with the journal editors. The cover letter is the first document in a manuscript file that is read by the editor and serves as a formal introduction to your communications with a journal. It also functions as an official record within the manuscript management system and a convenient way of transmitting essential information about the manuscript, authors(s), and regulatory standards. The cover letter is usually written by the corresponding author on behalf of all the contributing authors on the paper [20]. As you write your cover letter, you are also encouraged to use this opportunity to make suggestions for reviewers to the editor, to provide a better understanding of the field in which the study fits.

#### Common pitfalls and how to avoid them

Pitfalls can present themselves in a number of different ways throughout the publishing process (Table 2). The following is a discussion around some of the common pitfalls: *Choosing the wrong journal*. The process of choosing a journal to submit your work is an important decision. Choosing a journal requires careful consideration of details such as the aims and scope of the prospective journals. It is important to identify alignment between the goals of the journal and your work, so you can choose journals that will help you reach your intended audience. *Poor cover letter on journal submission*. As discussed previously, the cover letter is an opportunity to make a first impression. Spend time on writing a detailed cover letter. Drafting a thoughtful cover letter is a good way to avoid delays in the publishing process. An error free cover letter helps to enhance communications about your work, including the significance of the study which can help the editorial board make informed decisions. *Failure to follow target journal guidelines*. Be sure to review all journal submission guidelines carefully and refer to the submission guidelines often during the submission preparation process. Budget your time wisely to address all submission guidelines appropriately. Errors can cause unnecessary delays. *Not responding appropriately to feedback*. When you receive feedback from reviewers, create a plan to help you manage your time wisely with this final stage of the process. Having a plan can help you address reviewer feedback in a thoughtful and timely manner. Being aware of these pitfalls can help researchers avoid common errors that could potentially lengthen or complicate the overall process of getting your work published. Paying attention to such details can better position researchers to achieve their goal of successfully publishing high-quality science that will advance the field [21].

**Table 2:** Common pitfalls and how to avoid them

Pitfall	How to Avoid Pitfall
Choosing the wrong journal	• Always look at the aims and scope of prospective journals • Pick a journal that will help you reach your intended readership
Poor cover letter on journal submission	• Spend time writing a detailed cover letter • Use the cover letter to highlight the significance of the study
Failure to follow target journal guidelines	• Review all journal submission guidelines carefully • Budget your time wisely to address all submission guidelines appropriately
Misinterpret the pre-submission inquiry	• Understand that pre-submission inquiries are not publishing agreements (a nod to submit is not a guaranteed of acceptance) • Use pre-submission inquiries as an opportunity to talk to editors in the field about your work
Underestimating the publishing process	• Manage your time wisely around the submission and revision phases • Address submission and revision requirements/concerns in a timely manner
Not responding appropriately to feedback	• Do not take reviewer comments personally • See reviewer feedback as an opportunity to critically evaluate your work • Address all feedback but understand that you are not required to make all of the suggested changes • Do not waste time trying to identify specific reviewers

## Conclusions

Scientific publishing is an integral part of academic careers. In this article, we reviewed the viewpoints and advice from editors-in-chief for cohorts in NIH-ASCB Accomplishing Career Transition program, which are also applicable to postdocs and junior faculty in biological/biomedical sciences. At first, designing a targeted plan for publications sets yourself up for success. “Writing as you go” could be the mantra when conducting research projects. Specifically, the organization, data, and figures are the key elements of a high-quality paper. Finally, avoiding common pitfalls throughout the submission process increases the chances of research works being published.

## Abbreviations

ACT: Accomplishing Career Transition
ASCB: American Society for Cell Biology
IPERT: Innovative Programs to Enhance Research Training
JCB: Journal of Cell Biology
MBoC: Molecular Biology of the Cell
NIH: National Institutes of Health
